# Rare resected eight cases of duodenal adenocarcinomas

**DOI:** 10.1016/j.ijscr.2021.106384

**Published:** 2021-09-07

**Authors:** Atsushi Nanashima, Yukinori Tanoue, Naoya Imamura, Masahide Hiyoshi, Koichi Yano, Takeomi Hamada, Takahiro Nishida, Kengo Kai, Yasuto Suzuki, Yuichiro Sato, Koji Nakashima, Ayumu Hosokawa, Takeshi Nagayasu

**Affiliations:** a Division of Hepato-Biliary-Pancreas Surgery, Department of Surgery, University of Miyazaki Faculty of Medicine, Japan; b Division of Surgical Oncology, Nagasaki University School of Biomedical Sciences, 1-7-1 Sakamoto, Nagasaki 852-8501, Japan; c Department of Diagnostic Pathology, University of Miyazaki Hospital, 5200 Kihara, Kiyotake, Miyazaki 889-1692, Japan; d Department of Clinical Oncology, University of Miyazaki Hospital, 5200 Kihara, Kiyotake, Miyazaki 889-1692, Japan

**Keywords:** Duodenal adenocarcinoma, Combined resection, Adjuvant chemotherapy

## Abstract

**Introduction:**

Duodenal adenocarcinoma is a rare malignancy; recently, it has been found to be accompanied by operative indications.

**Methods:**

Nine consecutive rare cases were diagnosed with duodenal carcinoma (DC), in which clinicopathological characteristics were retrospectively examined. Age was ranged over middle-aged males and females. No clinical onset with severe symptoms was observed, and the specific treatment for accompanied diseases or habits was not found.

**Outcomes:**

One case of two T1 stage DCs that underwent pancreas-sparing duodenectomy. Stage II DC was diagnosed in three cases, and stage III DC was diagnosed in four cases. Pancreaticoduodenectomy (PD) mainly occurred in seven patients, and duodenectomy was limited in two patients. All operations were safely performed, and the postoperative course showed no severe morbidity. Histological findings showed R0 resection in eight cases and R1 at the retroperitoneal dissecting part in one case. Five patients with advanced-stage DC underwent adjuvant chemotherapy; however, four patients showed tumor recurrence within 12 months. With additional strong chemotherapy, eight patients survived up to 84 months, and one died of liver metastasis at 43 months after surgery. Three representative cases of mucosal invasion with widespread pancreas-sparing duodenectomy and advanced-stage DC cases undergoing duodenectomy or PD are shown.

**Conclusion:**

In the field of upper digestive tract surgery, duodenal adenocarcinoma and various applications of surgery or adjuvant chemotherapy for long-term survival are important.

## Highlights

• Duodenal cancer used to be rare disease and, however, we have increasingly experienced surgical intervention for duodenal cancers because it might be due to development of duodenal endoscopic examination.
• For early cancers, duodenal resection preserved surrounding organs such as a pancreas can be indicated, otherwise pancreaticoduodenectomy is necessary for advanced stage duodenal cancer located up to 3rd portion of duodenum.
• Patient prognosis can be expected by the novel adjuvant chemotherapy after curative duodenectomy.

## 1 Introduction

Duodenal carcinoma (DC), except duodenal papilla of Vater carcinoma, is a very rare digestive cancer; however, we have had the opportunity to experience patients with DC, indicating surgery in the last decade, as well as other reports, in Japan [1], [2]. The standard operative procedures may not be definitely decided; furthermore, effective chemotherapy has not been fully elucidated at present [1], [3]. Therefore, patient prognosis cannot be expected by the complete operation combined with surrounding organs. However, the recent imaging modality or the spread of periodic medical examination by advanced-quality endoscopy would provide an earlier stage of DC and endoscopic resection than previous periods [4], [5], [6], [7]. The development of adjuvant chemotherapy is also promising for DC. The clinico-pathological characteristics and patient outcome in DC has not been fully clarified and this rare case series seems to be unique.

We herein report 9 patients with DC who underwent radical operation with or without surrounding organs, such as the pancreas, at two academic institutes, as experienced by the principle author. We also discuss the operative applications and adjuvant treatments based on this series.

## 2 Methods

This study series were accordant with the Declaration of Helsinki established in the 1964, and every research study involving human subjects must be registered in a publicly accessible database of both University of Nagasaki and University of Miyazaki hospitals (ethical accept number; O-0568, 2019). The perioperative data of this case series was retrospectively and consecutively collected and examined at two above academic institutes without any criteria (summarized in Table 1). This study was set at the teaching academic hospital and data were collected by the long-term follow-up by our outpatient clinics and any patient's personal name, identification number were de-identified. The clinico-pathological diagnosis was according to UICC staging system. Any preoperative treatment intervention for DC were not performed in this series. All patients underwent curative operation with lymphadenectomy and anastomosis under general anesthesia and intensive care unit management after surgery at day 1. The operation was undergone by the primary author and a few experienced staff in co-authors at both institutes. Operative quality was controlled by the primary author in all operation.

**Table 1:** Patient demographics of duodenal cancer.

	Age	Gender	Location of DC	Abdominal symptom	Accompanied diseases	Prior operation	Smoking habit	Alcoholism	UICC TNM stage^a^	UICC stage^a^
1	52	M	2nd portion	Yes	Hypertension		No	No	t4b, n2	3b
2	67	F	2nd–3rd portion	No, health check			No	No	t1, n0	I
3	51	M	2nd portion	No, health check	Osler’s disease, colon polyposis, IPMN, pulmonary AVF	Splenectomy, colectomy	No	No	t1, n0	I
4	66	M	2nd portion	Yes			No	No	t3, n0	2a
5	65	M	2nd portion	Yes	Rectal cancer, diabetes	Abdominoperineal excision	No	No	t4b, n0	2b
6	73	M	2nd portion	Yes	Hypertension, Hepatolithiasis		Yes	Yes	t4b, n0	2b
7	70	M	2nd portion	Yes	Hypertension, cystic lung		Yes	Yes	t4b, n2	3b
8	58	F	4th portion	Yes			No	No	t3, n1	3a
9	71	F	2nd portion	Yes		Appendectomy	No	No	t4, n2	3b

M: male, F: female. IPMN; intraductal papillary mucin-producing neoplasm of pancreas. AVF; arterio-venous fistula, is; carcinoma in-situ.

a TNM; tumor-node-metastasis and UICC stage for small intestine adenocarcinoma [15].

## 3 Results

### 3.1 Summary of eight DC cases

Table 1 summarizes the clinicopathological features, operative procedures, adjuvant treatments and postoperative outcomes of patients. The gender was male in six cases and female in three cases. The disease-specific accompanying disease, lifestyle habits and abdominal symptoms with duodenal carcinoma were not remarkable. Two cases had early-stage carcinoma; however, the remaining 7 cases showed advanced-stage adenocarcinoma. Seven patients underwent stomach-preserving PD, and the remaining two patients underwent limited duodenal resection with mesentery for early- and advanced-stage carcinoma (Table 2). All patients safely underwent surgery, and case 2 alone had a blood transfusion. Seven patients showed advanced ulcerative tumors, and the remaining two patients showed flat elevated and polypoid findings in the specimens. The tumor size ranged between 2.6 and 7.5 cm and was relatively larger than that of small intestinal carcinoma. The eight cases had surrounding organ or mesenteric invasion, and the histological differentiation varied (Table 3). All patients but case 5 underwent R0 radical operations. Four patients had a postoperative pancreatic fistula. Oral intake was restarted within 7 days in eight of nine cases. No delayed gastric emptying or ileus was observed. Adjuvant chemotherapy was not administered in four cases, S1 in three cases and modified FOLFOX6 in two cases. All patients but case 4 are still alive, and four cases had a postoperative recurrence with distant metastasis.

**Table 2:** Operative results.

	Operation	Digestive reconstruction	Lymphadenectomy	Blood loss (mL)	Transfusion	Operating time (min)	Macroscopic finding	Size (cm)
1	SSPPD	PJ	D2	1150		742	Type3	6
2	DR, 2nd-4th portion	PBJ+DJ(EE)	D1	150			Flat elevated	2.8
3	SSPPD	PJ	D1	860	Yes	766	Polypoid	3
4	SSPPD	PJ	D2	490		507	Type 2	5.5
5	SSPPD	PJ	D2				Type 3	7.5
6	SSPPD	PJ	D2				Type 3	6
7	SSPPD	PJ	D2	340		650	Type 2	2.6
8	DR, 3rd–4th portion, transverse colectomy	DJ(ES)	D2	180		412	Type 2	6.8
9	SSPPD	PJ	D2	470		428	Type 3	4.2

SSPPD; subtotal stomach preserving pancreatico-duodenectomy, DR; duodenal resection. PJ; pancreatico-biliary jejunostomy, DJ; duodeno-jejunostomy. EE; end-to-end anastomosis, ES; end-to-side anastomosis. Regional dissection level [15].

R1; histologically cancer positive at the dissecting margin.

**Table 3:** Histological characteristics and patient outcomes.

	Specific histology	histological differentiation	Vessel infiltration	Curability	Postoperative complication	Oral intake (days)	adjuvant chemotherapy	Recurrence (month)	Prognosis (month)
1	Pancreas invasion	Moderately	ly2v3	R0	Nil	6	S1 (12mo.)	Peritoneum (12mo.)	Alive (39mo.)
2	Mucosal spread	Well	ly0v0	R0	Nil	7	Nil	None	Alive (84mo.)
3	Mucosal lesion with BD-IPMA	Well	ly0v0	R0	PF grade B*, IA	5	Nil	None	Alive (48mo.)
4	Mesocolon invasion	Moderately	ly2v3	R0	PF BL*	5	S1 (6mo.)	Liver (7mo.)	Cancer death (43mo.)
5	Duodenal stenosis, pancreas invasion	Well	ly0v3	R1**	Wound infection	4	S1 (6mo.)	None	Alive (30mo.)
6	Pancreas invasion	Papillary, mucinous	ly0v2	R0	PF BL*, wound infection	4	Nil	None	Alive (43mo.)
7	Dilated MPD, pancreas invasion	Moderately	ly1v1	R0	Ascites	3	mFOLFOX6 (4 cycles)	Liver (12mo.)	Alive (37mo.)
8		Moderately	ly0v0	R0	Chile leak	10	mFOLFOX6 (12 cycles)	Lung (2mo.)	Alive (10mo.)
9	Pancreas invasion	Medullary	ly1v1	R0	PF grade B	3	Not yet	None	Alive (1mo.)

B-IPMA; Branched type intraductal papillary mucinous adenoma of pancreas, MPD; main pancreatic duct, ly; lymphatic, v; venous, PF; pancreatic fistula, *Grade of postoperative pancreatic fistula was defined by the Internal study group (ISGPS) definition and grading [20], BL; biochemical leak, IA; intraabdominal abscess, ** retroperitoneal dissecting margin, mFOLFOX6; modified FOLinic acid, Fluorouracil and Oxaliplatin 6.

### 3.2 The representative cases

**Fig. 1:**
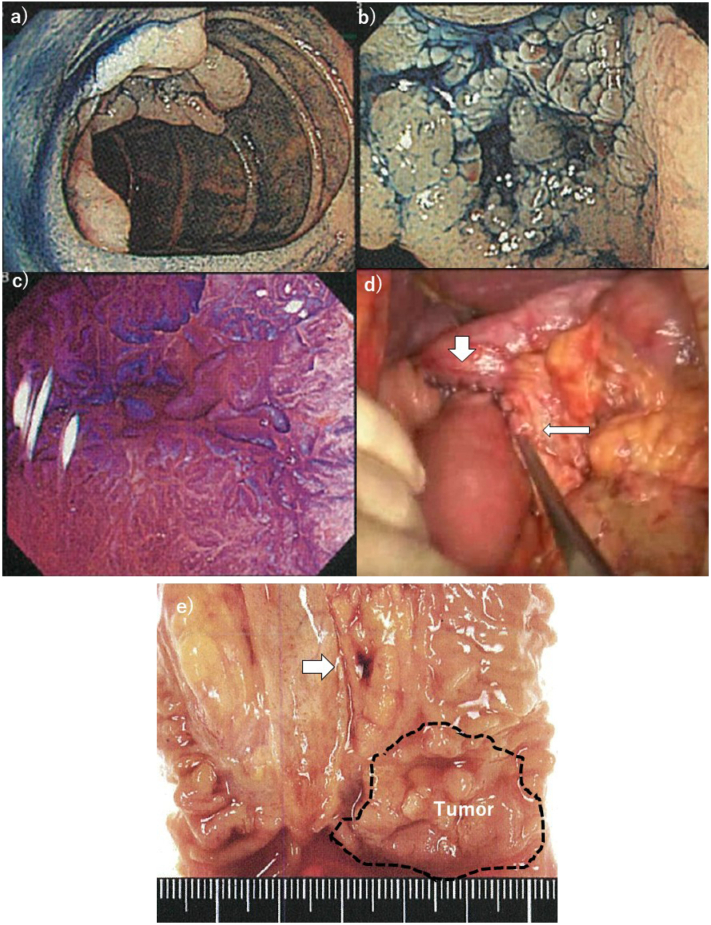
Findings of case 1. Endoscopic examination showing: a) a protruding tumor at the oral side adjacent to the duodenal papilla; b) an approximately 3 cm duodenal tumor spread to the anal side between the second and third portions; c) dye staining with no infiltrative finding, which shows adenocarcinoma by biopsy specimen; d) intestinal anastomosis after resection showing the end-to-end anastomosis between the duodenum and jejunum (thick arrow) and the end-to-side anastomosis of the pancreaticojejunostomy (thin arrow); and e) specimen showing a 3 cm protruding duodenal carcinoma adjacent to the duodenal papilla with R0 curative resection.

Case 2 represented an early-stage adenocarcinoma with superficial spread between the second and third portions of the duodenum. A 67-year-old woman had no clinical symptoms or signs but underwent a health-check-aim endoscopic examination. The oral side of the tumor was located adjacent to the duodenal papilla, and submucosal invasion was not observed on endoscopic examination (Fig. 1a–c) or accompanying ultrasonographic examination. As no lymph node metastasis was observed, duodenal resection between the second and fourth portions, including the duodenal papilla, was performed (Fig. 1d). The resected specimen showed 3 cm protruding duodenal adenocarcinoma adjacent to the duodenal papilla (arrow). The histological diagnosis showed well-differentiated adenocarcinoma invading the mucosa alone without lymphatic or venous infiltration, and R0 resection was achieved (Fig. 1e). The patient's postoperative course was uneventful, and she has been alive over 5 years without cancer relapse.

**Fig. 2:**
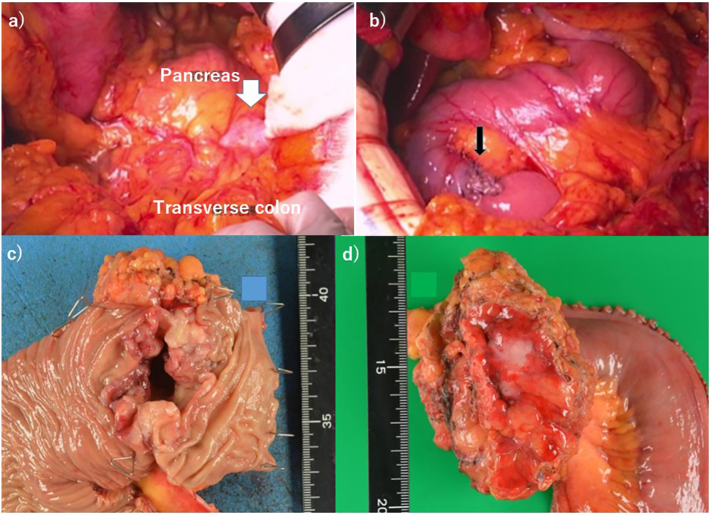
Operative findings showing: a) a DC at the fourth portion of the duodenum (white arrow) that infiltrated beyond the serosa and was located adjacent to the body of the pancreas body; b) duodenojejunostomy with functional end-to-end anastomosis (thin black arrow); c) specimen showing a 6 cm ulcerative type 2 cancer; and d) tumor invading the mesentery of the transverse colon beyond the serosa.

Case 8 was a 58-year-old woman with advanced duodenal carcinoma at the fourth portion of the duodenum who had epigastralgia and tarry stools. A 6.8 cm tumor was found at the fourth portion of the duodenum, without intestinal obstruction. The pancreas body and superior mesenteric artery (SMA) were adjacent to tumor invasion (Fig. 2a); however, duodenectomy at the third and fourth portions accompanied by transverse colectomy was performed. Anastomosis of the duodeno-(2nd portion)jejunostomy was performed (Fig. 2b). Tumors showed ulcerative tumors invading the serosa and colonic mesentery (Fig. 2c and d). Histological analysis showed moderately differentiated adenocarcinoma without lymphatic or venous infiltration, and R0 resection was achieved. Chyle leakage was observed by postoperative day 24. Twelve adjuvant cycles of modified FOLFOX6 (40% decrease in full dose) were administered, and no remarkable recurrence was observed for 10 months.

**Fig. 3:**
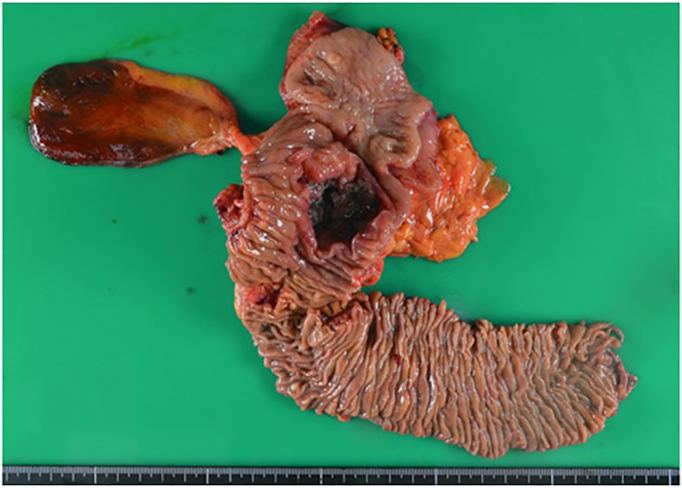
Specimen showing a 4.2 cm type 3 ulcerative carcinoma near the papilla of Vater, which invaded the parenchyma of the pancreatic head parenchyma.

Case 9 was a 71-year-old woman with advanced duodenal carcinoma at the second portion of the duodenum who had epigastralgia, gastric distension and obstructive jaundice. The 4.2 cm tumor invaded the pancreatic head, and therefore, pancreaticoduodenectomy (PD) was performed (Fig. 3). Histologically, medullary differentiated adenocarcinoma with mild lymphatic and venous infiltration was observed, and R0 resection was achieved. A pancreatic fistula was observed after the operation and was conservatively cured. Adjuvant chemotherapy was denied by the patient; however, no remarkable recurrence was observed for 12 months.

## 4 Discussion

The clinicopathological features, surgical treatment strategy and prognosis of duodenal carcinoma have not been well elucidated because of its rarity, and its actual prevalence or pathogenesis might be unclear even now. Our study results showed the relatively good patient outcomes accompanied with curability. According to Alwmark et al., the prevalence of duodenal carcinoma was 0.3% of all digestive tract carcinomas and 25-45% of small intestinal carcinomas; however, this report was published in 1980 [8]. Recent data have not been fully elucidated. In Western reports, DC is not rare, and many surgeries have been carried out [9], [10], [11], [12]. Longer survival and many 5-year survivors might be achieved at present [9], [13]. Reports regarding radical resection and the better prognosis of surgical resection compared to non-operative cases have been recently clarified [14]. The staging classification of DC was defined by the present 8th UICC staging system [15]. In the last decade, the diagnosis of epithelial neoplasia of the duodenum has been increased by endoscopic examination in Japan; therefore, the development of this diagnostic tool is considered to be a reason for increased findings [4], [5], [6], [7]. Regardless, according to finding DC at an earlier stage that can be locally located in the duodenum, radical surgery seems to be more frequent than in previous periods according to reports and our experiences [1], [2], [9], [10], [11], [12], [13], [14], [15]. In fact, our present series of nine patients showed that the first curative case was found in 2014; therefore, preliminary evaluation of surgical treatment for DC was examined in this report.

According to previous reports, the predominant age of DC was middle or high age, but there was no sex tendency [8], [16]. In the present series, many DC cases were found at an advanced stage, which was located on the oral side from the second portion, but DC at the duodenal bulb was not observed. Kobayashi's recent report showed that four of seven (57%) DCs were located in the bulb (first portion), and distal gastrectomy was performed, which was found at a relatively early stage [1]. Anemia due to tumor bleeding was the main clinical symptom, and such a clinical symptom might easily appear due to pyloric outlet disturbances. Due to the tumor location, the time of appearance of symptoms might be delayed in comparison with the progression of the tumor, and the operative procedure and lymphadenectomy area seem to be different. In our series, the main location was around the second portion near the papilla of Vater; therefore, the clinical onset of symptoms might be delayed, and tumors might be found in an advanced stage. As a result, PD was mainly performed. In a recent Western series, patients with DC undergoing PD and duodenectomy were compared. The overall survival was not significantly different, although patients undergoing PD had higher morbidity [9], [10], [13]. However, it depends on the extension of the tumor into the parenchyma of the pancreas [17], [18]. Unnecessary PD was avoided if the tumor did not invade the pancreatic side. Interestingly, complete obstruction was not observed. Otherwise, two cases were found to be early-stage (T1) carcinomas due to routine endoscopic health checks. Duodenal adenomas or non-epithelial tumors can be found at present, and endoscopic mucosal resection or laparoscopic-assisted endoscopic surgery can be developed [6], [7]. In our series, the depth of tumor infiltration was accurately diagnosed by endoscopic ultrasonography (EUS), and one patient underwent pancreas-sparing duodenectomy (case 2). However, another patient underwent excessive PD because the accompanying branch-type IPMN was located in the pancreatic head. As a result, IPMN showed a low-grade adenoma, which did not need to be resected. The advanced DC in case 8 was located at the fourth portion of the duodenum. In this case, combined distal pancreatectomy and SMA resection was expected. Although this combined resection could be avoided, mesocolon invasion was remarkable, necessitating the resection of it and the transverse colon together. In this case, the diseased duodenum was not completely obstructed, and no vomiting was observed. This DC seemed to be close to the jejunal carcinoma, and the lymphadenectomy area was around the jejunal artery or venous branches 1 to 3 and middle colonic vessels adjacent to the SMA. López-Domínguez et al. reported that patients with DC in the third or fourth portion could undergo segmental duodenectomy with good prognosis [13]. In our series, although some postoperative complications occurred, the short-term surgical results were nevertheless good after aggressive surgery; thus, the operative risk might not be severe or so high. Postoperative nutritional status was also well maintained (detailed data are not described in the text) in all cases.

In previous reports, some associated clinicopathological factors with respect to patient prognosis were older age, preoperative albumin status, gastric outlet obstruction, surgical resection margin status (R0), node metastasis, poor differentiation, perineural invasion, lymphovascular infiltration, tumor stage and adjuvant chemotherapy [1], [2], [14], and the independent factors were supposed to be tumor stage and R0 resection status. In our series, most patients were middle- to high-age patients, but preoperative nutritional status was maintained by our prehabilitation or nutritional support team's protocols. All but one patient (R1) underwent R0 resection. Nevertheless, in advanced stages III and IV, the node metastasis status was not high, and lymphatic or vessel infiltration was mild in many patients. Histological findings showed no poor differentiation or perineural infiltration. Thus, all 8 patients underwent aggressive surgery in good condition. Adjuvant chemotherapy has not been fully established, and we should select a protocol for gastric or small intestinal carcinomas (equivalent to the colorectal carcinoma protocol) [3]. According to previous reports, DCs located near the bulb might undergo an S1-based gastric cancer protocol or an oxaliplatin-based multidrug regimen for colorectal protocols [1]. Recently, JCOG1502C performed a randomized phase III trial of adjuvant chemotherapy for patients with small bowel adenocarcinoma (surgery alone vs. capecitabine and oxaliplatin) [3]. Western reports also showed that adjuvant chemotherapy led to prolonged survival of patients with DC [9], [12]. In our series, except for stage I cases, the patients received S1 until 2017, and the modified FOLFOX6 regimen was induced at present. Although some had tumor recurrence after adjuvant chemotherapy, the patients tolerated continuous multidrug chemotherapy well; eventually, all patients had a longer survival of up to 43 months in patients with advanced DC. As the expected adjuvant treatment and prehabilitation program, aggressive R0 resection with extended lymphadenectomy can be aimed at patients with DC.

Careful and frequent follow-up in patients with DC is necessary because of concerns about multiple cancers occurring in the remnant duodenum or small intestine, and longer survivors exist in Western reports at present [13]. By considering these issues, adequate bowel anastomosis considering the easy endoscopic route after surgery in addition to extracorporeal imaging examinations, such as a CT, can be considered. In this series, we attempted to perform bowel anastomosis with surgical ingenuity, as shown above. Although we reported nine cases, a cohesive cohort study has not yet been fully collected [9], [10], [11]. Nationwide or international cohort studies at multiple cancer institutions are expected.

In conclusion, we report nine rare cases of DC located between the second and fourth portions of the duodenum in middle- to high-age patients. Diseases included two cases of T1 stage and six of advanced-stage DC. Most patients underwent R0 resection, but one underwent R1 resection; good outcomes and long-term survival might be provided by adjuvant chemotherapy. Careful postoperative follow-up was performed. Through our experiences of such rare advanced malignancies, we recognized that comprehensive collaboration among surgeons, endoscopists, chemotherapy physicians and pathologists is important. The multi-institutional study is necessary because of disease rarity.

## Human rights

All procedures were performed in accordance with the ethical standards established in the 1964 Declaration of Helsinki and its later amendments.

## Informed consent

Informed consent was obtained from eight patients described in this report.

We state that the work has been reported in line with the PROCESS 2020 (www.processguideline.com) criteria and cite the paper by Agha RA et al. [19]

## Provenance and peer review

Not commissioned, externally peer-reviewed.

## Declaration of competing interest

Authors declare no conflicts of interest. No financial support for this study.
